# Acute and Delayed Effects of Time-Matched Very Short “All Out” Efforts in Concentric vs. Eccentric Cycling

**DOI:** 10.3390/ijerph18157968

**Published:** 2021-07-28

**Authors:** Daniel Boullosa, Boris Dragutinovic, Jan-Philip Deutsch, Steffen Held, Lars Donath, Wilhelm Bloch, Moritz Schumann

**Affiliations:** 1 Integrated Institute of Health, Federal University of Mato Grosso do Sul, 79070-900 Campo Grande, Brazil; 2 Institute of Cardiovascular Research and Sports Medicine, Department of Molecular and Cellular Sports Medicine, German Sport University, 50933 Cologne, Germany; b.dragutinovic@dshs-koeln.de (B.D.); w.bloch@dshs-koeln.de (W.B.); m.schumann@dshs-koeln.de (M.S.); 3 Intervention Research in Exercise Training, German Sports University, 50933 Cologne, Germany; janphilip.deutsch@web.de (J.-P.D.); s.held@dshs-koeln.de (S.H.); l.donath@dshs-koeln.de (L.D.)

**Keywords:** high-intensity interval training, sprint interval training, exercise-induced muscle damage, post-activation performance enhancement, muscle fatigue

## Abstract

Background: To the authors’ knowledge, there have been no studies comparing the acute responses to “all out” efforts in concentric (isoinertial) vs. eccentric (isovelocity) cycling. Methods: After two familiarization sessions, 12 physically active men underwent the experimental protocols consisting of a 2-min warm-up and 8 maximal efforts of 5 s, separated by 55 s of active recovery at 80 rpm, in concentric vs. eccentric cycling. Comparisons between protocols were conducted during, immediately after, and 24-h post-sessions. Results: Mechanical (Work: 82,824 ± 6350 vs. 60,602 ± 8904 J) and cardiometabolic responses (mean HR: 68.8 ± 6.6 vs. 51.3 ± 5.7% HRmax, lactate: 4.9 ± 2.1 vs. 1.8 ± 0.6 mmol/L) were larger in concentric cycling (*p* < 0.001). The perceptual responses to both protocols were similarly low. Immediately after concentric cycling, vertical jump was potentiated (*p* = 0.028). Muscle soreness (VAS; *p* = 0.016) and thigh circumference (*p* = 0.045) were slightly increased only 24-h after eccentric cycling. Serum concentrations of CK, BAG3, and MMP-13 did not change significantly post-exercise. Conclusions: These results suggest the appropriateness of the eccentric cycling protocol used as a time-efficient (i.e., ~60 kJ in 10 min) and safe (i.e., without exercise-induced muscle damage) alternative to be used with different populations in future longitudinal interventions.

## 1. Introduction

High-intensity training (HIT) is a training modality which consists of short bouts of high-intensity exercise interspersed with active or passive rest intervals. HIT is generally considered to be an efficient method for health and performance development and has been shown to induce improvements in both aerobic and anaerobic working capacities, cardiovascular health, and neuromuscular performance after short periods of time in various populations [1]. Sprint interval training (SIT) is a modification of HIT that has been introduced to further reduce time commitments and consists of brief “all out” efforts at supramaximal intensities [2]. Interestingly, despite a much lower overall exercise duration (i.e., 20–30 s per bout), the health and performance benefits do not relevantly differ between SIT and HIT regimens [3]. However, traditional SIT modalities have previously been criticized because of the excessive physical and psychological demands [4,5]. For this reason, recent studies have proposed modified SIT by further shortening both the duration of the whole session and each sprint (≤10 s), thus showing rapid aerobic and anaerobic adaptations with less time-commitments and reduced fatigue [6,7].

Eccentric cycling has recently gained interest as a promising modality due to reduced metabolic demands associated with lower muscle activation for a given torque output as compared to concentric cycling with similar cadences [8]. This specific characteristic could be interesting for diverse populations of athletes or patients under specific circumstances (e.g., coronary and pulmonary patients). However, when non-adequately designed, eccentric cycling may result into altered autonomic nervous system responses that could adversely reduce the safety of patients [9]. Most studies on eccentric cycling have been completed at continuous submaximal intensities, with few studies examining eccentric HIT [10,11,12]. For instance, an initial study [10] evaluated the reliability of 6-s eccentric “sprints” over four days and suggested that two familiarization sessions are necessary for acceptable reliability of power outputs at different cadences. Subsequently, Lipski et al. [11] compared different eccentric continuous and HIT protocols and observed that the greatest VO_2_ and perceived exertion were observed during the supramaximal protocol of 10 × 1-min bouts at 150% of concentric peak power output (PPO). More recently, Mavropalias et al. [12] have suggested that high- and low-intensity eccentric cycling with matched mechanical work induced similar decreases in muscle function, but the delayed onset of muscle soreness (DOMS) was greater after high-intensity eccentric cycling. Although this preliminary research shows promising results, there is still an important lack of studies examining eccentric cycling HIT protocols and, more specifically, protocols with very short “all out” efforts which would allow the achievement of higher mechanical workloads in very short sessions. Thus, the precise characterization of the acute responses of eccentric protocols with very short “all out” efforts would be important to elucidate the potential of this novel exercise alternative in terms of dose-response as well as possible risks and benefits [13].

Despite the reported benefits of eccentric cycling, possible risks also need to be considered [14]. Most commonly, eccentric cycling is accompanied by exercise-induced muscle damaged (EIMD) which is characterized by muscle soreness, inflammation, and loss of functional capacity, such as strength loss and decreased proprioceptive function [14]. It is well accepted that eccentric contractions are more prone to inducing EIMD than concentric work, however recent evidence suggest that this may be attributed to muscle unaccustomedness and not to eccentric exercise per se [15]. In this regard, there are a number of confounding factors other than mechanical stress that may be associated to the etiology of EIMD after eccentric contractions, including the relative exercise intensity [16] and the metabolic milieu [17,18]. Moreover, the relationship between loss of functional capacity and muscle damage may be confounded by the low-frequency fatigue associated with eccentric exercise [16], indicating that force loss is not necessarily related to changes in markers of muscle damage and soreness in the short-term [19]. This picture is further complicated when considering the limited knowledge of the disruption of sarcomeres during eccentric actions and their effects on EIMD symptoms [20]. More recently, a novel hypothesis states that delayed onset of muscle soreness (DOMS) may be the result of an acute compression axonopathy which may be subsequently enhanced by inflammation [21]. This complex picture reinforces the need to look for diverse markers of EIMD, apart from the most used creatine kinase (CK) [22], to better understand the acute and delayed responses of EIMD symptoms. Among others, novel markers of proteostasis such as collagenase matrix metalloproteinase 13 [MMP-13] and BCL2-associated athanogene 3 [BAG3] may add relevant information as they are involved in different mechanisms than CK after EIMD. Thus, MMP-13 is a very potent extra cellular matrix (ECM)-degrading enzyme [23], which is involved in muscle regeneration through its role in myoblast migration [23]. In addition, BAG3 is a chaperone-assisted selective autophagy component acting at the Z-disk level [24]. Strenuous muscle contractions were previously shown to exhibit significant reductions in muscle concentrations of BAG3 24 h post-exercise [25]. Therefore, the simultaneous evaluation of diverse muscle damage markers, muscle soreness and swelling, and functional capacity, would allow for a better characterization of the acute and delayed effects of eccentric cycling with very short “all out” efforts.

The aim of the present study was to describe and compare the metabolic, perceptual, and mechanical responses of an eccentric vs. concentric time-matched cycling sessions with very short “all out” efforts. Furthermore, we aimed to examine the acute effect of these protocols on selected measures of muscle damage and functional performance. Our hypotheses were that the internal load parameters of the eccentric protocol (e.g., heart rate, blood lactate) would be lower than that of concentric protocol, and that possible detrimental effects of EIMD (e.g., muscle soreness, loss of muscle force) would be greater following eccentric compared to concentric “all out” efforts.

## 2. Materials and Methods

**Figure 1:**
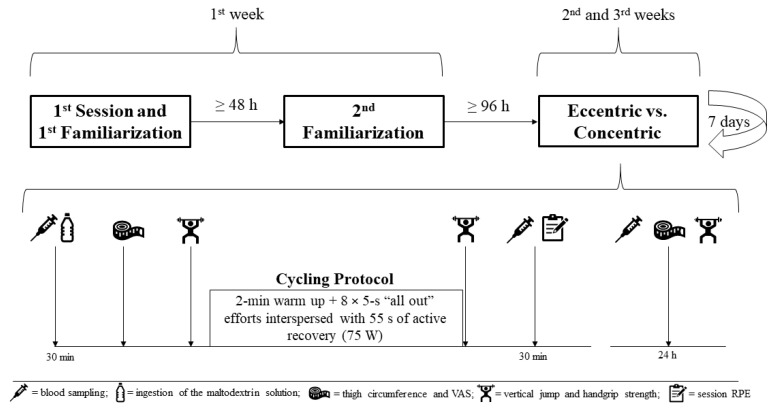
Study design.

This study consisted of six laboratory visits over three weeks, with the activities completed on each visit as follows: (1) anthropometric evaluations, graded cycling test and initial familiarization with eccentric cycling; (2) familiarization of the concentric and eccentric protocols, including functional performance testing and perceptual scales; and (3–6) experimental interventions consisting of concentric and eccentric protocols and a 24-h follow-up measurement in a randomized order. The first and second visits occurred in the same week with a minimum of 48 h between sessions, while the experimental sessions and their corresponding recovery assessments were carried out one week apart. Measurements included functional performance (vertical jump and handgrip strength), perceptual measures (rating of perceived exertion [RPE], feeling scale [FS], visual analogue scale [VAS] and session RPE [sRPE]), thigh circumference, and blood markers of muscle damage (CK, MMP-13, and BAG3). To avoid circadian influences, all evaluations were completed at the same time of the day, between 7:30 and 9:30 a.m. The study design is presented in Figure 1.

Twelve physically active young men (age: 23.4 ± 2.8 years; height: 183 ± 6 cm; body mass: 76.6 ± 7.8 kg; body fat percentage: 10.8 ± 3.5%), most of whom were involved in recreational endurance sports, participated in this study. A minimum of 6 subjects would be required to account for differences between concentric and eccentric cycling protocols following an a priori power calculation assuming a Cohen’s d = 2, an α = 0.05, and a power = 0.95 (v. 3.1.9.4, G*Power software, Heinrich Heine Universität, Düsseldorf, Germany), in agreement with previous literature [26]. The inclusion criteria consisted of being physically active, not presenting any medical condition that would contradict the performance of strenuous exercise, not using any substance or medication during the time of the study, and no practice of regular strength training. All subjects were asked to maintain nutritional habits throughout the intervention period and to avoid strenuous exercise 24 h prior to each laboratory testing session.

Subjects’ physical work capacity was assessed by a graded cycle ergometer (SRM Ergo, SRM Training Systems GmbH, Leipzig, Germany) test. The test consisted of an initial 2 min at 100 W, with subsequent load increments of 15 W every 30 s. Subjects were required to maintain a pedaling frequency of ~80 rpm throughout the test. The respiratory gas exchange was continuously monitored via a validated metabolic cart (Metamax 3b, Cortex Biophysics, Leipzig, Germany). Subjects were verbally encouraged until volitional exhaustion. Peak oxygen uptake (VO_2_peak) averaging the VO_2_ recorded over the final 30 s was validated after attainment of ≥ 90% of the age-predicted maximum heart rate (HRmax) [27], a respiratory exchange ratio (RER) ≥ 1.10, and a RPE ≥ 19 (Borg’s 6–20 scale). Similarly, the PPO was determined as the average of the values recorded during the final 30 s of the ramp test.

Two familiarization sessions with the eccentric ergometer (Cyclus2 Eccentric Trainer, RBM Elektronik-Automation GmbH, Leipzig, Germany) in a standard seated position were deemed sufficient for obtaining reliable mechanical responses in “sprint” eccentric cycling at 80 rpm [10], and probably providing some protection to avoid a exacerbate EIMD as observed in pilot testing, based on the repeated bout effect (RBE) [15]. The initial session took place 5 min after cessation of the ramp test. This session consisted of 2 min at ~75 W and ~80 rpm, followed by 3 efforts of 5 s each aiming to reach the PPO previously recorded in the ramp test (~414 ± 58 W). When subjects were not able to complete an effort correctly (e.g., losing contact with the pedal), an additional bout was added.

Forty-eight to 72 h following the ramp test, subjects reported to the laboratory for a second familiarization session. First, subjects were familiarized with the visual analogue scale (VAS) and procedures for the identification of DOMS. Subsequently, a short warm-up on the concentric cycle ergometer (2 min at 75 W, with ~80 rpm) was performed, and was followed by 3 “all out” efforts of 5 s interspersed with 55 s of active recovery at the same load as in the warm-up. After 3 min of rest, the same procedure was performed on the eccentric cycle ergometer. When subjects failed to complete an effort in the eccentric mode, an additional bout was added. Before and during these “sprint” bouts, subjects were also familiarized with perceptual scales. Finally, after a brief rest of 3 min, subjects completed 3–4 attempts of all the functional evaluations including vertical jumping and handgrip testing in the same order as that of subsequent evaluations. Randomization for the next experimental sessions was carried out at the end of this session by drawing lots.

The protocols were designed based on protocols used in previous studies [10,28] as well as on pilot testing. Both protocols commenced with a submaximal 2-min warm-up at 75 W and a cadence of ~80 rpm, followed by 8 × 5 s “all out” efforts and 55 s of active recovery with the warm-up load. In the eccentric protocol, subjects were instructed to break with maximal effort against the backward crank movement at ~80 rpm (isovelocity) as variable (increasing) cadences are not possible in the cycle ergometer used (Cyclus2 Eccentric Trainer, RBM Elektronik-Automation GmbH, Germany). In the concentric protocol, subjects were able to increase their cadences during the sprinting bouts (isoinertial), as normally occur in concentric cycling SIT of different durations. This is an important difference with a previous study by Green et al. [26] who used isokinetic cycle ergometers to evaluate differences in torque, power, and muscle activation between 10 s maximal efforts at different cadences in concentric vs. eccentric modes. We selected the isoinertial mode in concentric cycling because it is the most commonly used in sport and exercise settings. Total work (Joules, power × time) for each session was calculated in a custom-made Excel spreadsheet, based on the data provided by the cycle ergometers software.

Heart rate was continuously recorded during protocols (H7, Polar Electro Oy, Finland). In addition, peak blood lactate concentrations were determined 2 min after exercise cessation (i.e., immediately after jump evaluations) from capillary blood obtained from the earlobe (EBIOplus, EKF Diagnostic Sales, Magdeburg Germany).

Measures of exertion and mood were monitored during protocols with RPE (6–20 Borg’s scale) [29] and FS [30] before and after the 2nd, 4th, 6th, and 8th bouts, as well as following the final bout, following standard procedures. Additionally, the sRPE (CR-10 scale) was collected 30 min after the end of the protocols [31].

Vertical jumping capacity was evaluated through the countermovement (CMJ) test on a force plate at 500 Hz (Quattro jump, Kistler, Switzerland) before (pre), immediately after (post), and 24-h post-loading. During the test, the arms were placed on the hips and the subjects were encouraged to jump “as high as possible”, while allowing a free selection of the countermovement depth [32]. This test was completed three times after warming up, and two times immediately after the protocols, with a minimum of ~15 s between attempts. The best jump in terms of jumping height was selected for further analyses. Jumping height was calculated as the difference between the position of the center of mass at takeoff and the apex (cm). Peak power (PP) was recorded at the end of the concentric phase (W·kg^−1^). Vertical stiffness (Kvert) was calculated and defined as the maximum force divided by the distance covered during the countermovement, normalized by body mass (N·m^−1^·kg^−1^). These parameters were calculated in a custom designed Excel spreadsheet after exporting the raw force-time data from the corresponding software [32].

Handgrip strength was assessed at pre, post, and 24-h post-loading, to monitor possible fatigue in the hands and forearms associated with the high force needed to fix the body position during sprinting bouts in the ergometers used. This is an important consideration as most previous studies with eccentric cycling were performed with more comfortable recumbent ergometers. Maximum handgrip strength (kgf) was determined as the highest recorded force of three attempts with a validated dynamometer (JAMAR, Patterson Medical, Canada) in the seated position with the elbow flexed at 90° [33]. A recovery period of 1 min was used between attempts. Limb dominance for dynamic handgrip testing was determined following previous recommendations [34].

Venous blood samples were drawn from the antecubital vein before (pre) as well as 30 min and 24 h after the protocols. The sampling at 30 min post loading cessation was chosen to allow for diffusion of damage-associated markers from muscle to blood. Prior to the eccentric and concentric protocols, the subjects reported to the laboratory in a fasting state and were provided with a maltodextrin solution (0.35 g·kg^−1^ body mass mixed with 6 mL·kg^−1^ body mass) after the first blood sampling. Blood samples were collected in vacutainer tubes (BD-Belliver Industrial Estate, Plymouth, UK). The serum separation tubes were stored for 15 min at room temperature, after which they were centrifuged at 3500 rpm for 10 min at 4 °C (Heraeus Multifuge 3 L-R, Kendro Laboratory Products, Newton, MA, USA). Immediately after centrifugation, serum was separated into 1 mL aliquots (Sarstedt, Nümbrecht, Germany) and stored at −80 °C for further analyses.

Basal levels of C-reactive protein (CRP) were determined with an enzymatic kinetic assay method (Roche Diagnostics, Mannheim, Germany) using a Hitachi 912 Automatic Analyzer (Roche Diagnostics) to ensure the absence of any inflammatory or infection processes the days of evaluations during winter. Markers being reflective of muscle damage included CK, as well as MMP-13 and BAG3. The MMP-13 is considered to be a very potent extra cellular matrix (ECM)-degrading enzyme [23], which is also involved in muscle regeneration through its role in myoblast migration [23]. The BAG3 is a chaperone-assisted selective autophagy (CASA) component acting at the Z-disk level [24]. Serum concentrations of MMP-13 and BAG-3 were assessed using ELISA kits (EHMMP13, Thermo Fisher Scientific, USA and Novus Biologicals, USA, respectively), according to the manufacturers’ protocol. Serum concentrations of CK were assessed by an enzymatic kinetic assay method using an automatic analyzer (Hitachi 912, Roche Diagnostics, Germany).

Lower limb pain associated with DOMS was assessed by means of a VAS with an accuracy of 100 mm [35]. For this assessment, subjects reported in the seated position: (1) the perceived pain after palpation with 3 fingers in the middle lateral part of the vastus lateralis muscle, between the trochanter and the patella of the dominant leg; and (2) perceived lower limbs pain after standing up and seating on a chair, two consecutive times, as fast as possible. Touching and maneuver values were averaged and used for evaluation of pain-associated DOMS. All of these procedures were performed by the same investigator in all of the evaluations.

Thigh circumference was considered to be a surrogate for muscle swelling and assessed with a flexible tape to the nearest cm, at the same point of the VAS assessment that was marked with a permanent pencil during the first visit in order to avoid differences between days.

Normality of distribution was assessed using the Shapiro-Wilk test. The following data was not normally distributed and consequently log-transformed prior to analysis: VAS, CK, and MMP13. Mechanical and cardiometabolic characteristics of the two protocols were compared using a Student’s paired *t* test. Within- and between-condition differences for all other variables were assessed using repeated measures of ANOVA. Reproducibility was assessed using intraclass correlation coefficients (ICC) and within-subject coefficient of variation (CV). In addition, effect sizes (Cohen’s d) were calculated using mean differences between baseline and each time point. The Pearson product moment correlation coefficient (r) was used to verify the relationships between dependent variables and their %Δ. The level of significance for all tests was set at *p* < 0.05.

## 3. Results

### 3.1. Graded Exercise Test

Subjects exhibited a VO_2_peak of 61.0 ± 8.4 mL·kg^−1^·min^−1^ with a PPO of 414 ± 58 W in the ramp test.

### 3.2. Comparisons between Protocols

Internal and external session load parameters of the two protocols are provided in Table 1. All mechanical and cardiometabolic responses were statistically greater (*p* < 0.001) in the concentric protocol when compared to the eccentric protocol.

**Figure 2:**
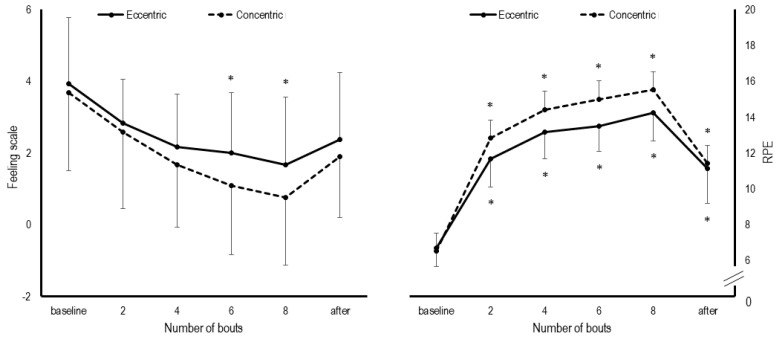
Differences in the Feeling Scale and Rating of Perceived Exertion (RPE) between protocols. * *p* < 0.05 compared to baseline.

The evolution of perceptual responses during the cycling protocols are shown in Figure 2. The RPE statistically increased throughout all time points during both concentric (d = 2.624 to 6.981; *p* < 0.05) and eccentric protocols (d = 2.849 to 5.658; *p* < 0.05). In the eccentric protocol, statistical increases in the FS were observed following the 6th (*p* = 0.030, d = 1.261) and 8th bouts (*p* = 0.016, d = 1.438). In the concentric one, FS remained statistically unaltered throughout the loading (d = 0.506 to 1.438; *p* > 0.05). There was no statistical difference for sRPE between concentric and eccentric sessions (5.2 ± 1.7 vs. 4.4 ± 1.5, *p* = 0.08, d = 0.473).

Functional performances at different time points in both conditions are presented in Table 2. Their reliability values were excellent (ICC > 0.9; CV < 10%) in all cases, except for Kvert with an ICC = 0.56 and a CV = 18.8%. The jumping height statistically increased immediately after concentric (6.1 ± 6.1%, *p* = 0.028, d = 0.364) but not eccentric cycling (+3.2 ± 3.8%, *p* = 0.080, d = 0.172). Jumping height was not different from the baseline at 24 h following the concentric (*p* = 0.898, d = 0.103) and eccentric protocols (*p* = 0.428, d = 0.131).

**Figure 3:**
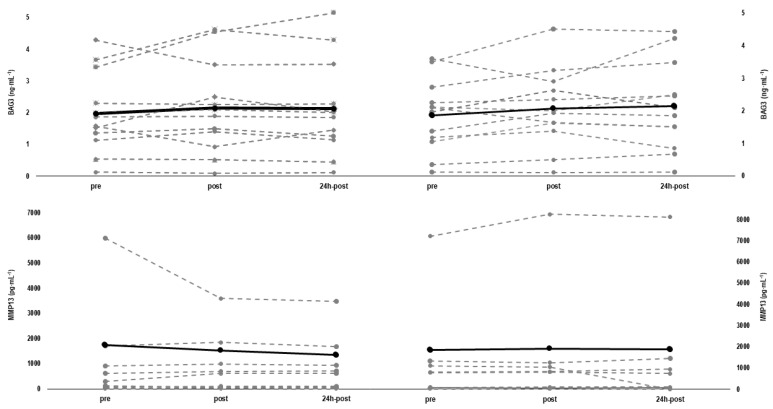
Individual relative changes and mean values (solid line) for collagenase matrix metalloproteinase 13 (MMP-13) (*n* = 9) and BCL2-associated athanogene 3 (BAG3) (*n* = 12) from baseline (pre), immediately after (post), and 24 h after (24 h-post) eccentric cycling (left panel) and concentric cycling (right panel).

All subjects showed basal values ≤ 0.3 mg/L for CRP on both testing days. Baseline values and their corresponding reliability measures of BAG3, MMP-13, CK, thigh circumference, and VAS are provided in Table 3. The individual changes of MMP-13 and BAG3 concentrations between protocols and moments are shown in Figure 3. Three individuals were not included for comparisons of MMP-13 (*n* = 9) because their values were out of range. Concentrations of BAG3 remained statistically unaltered throughout both the concentric (∆pre-post: *p* = 0.532, d = 0.174, and *p* = 0.145, d = 0.226) and eccentric protocols (∆pre-post: *p* = 1.000, d = 0.132, and ∆pre-24 h-post: *p* = 1.000, d = 0.108). Similarly to BAG3, concentrations of MMP13 remained statistically unaltered following both the concentric (∆pre-post: *p* = 1.000, d = 0.040, and ∆pre-24 h-post: *p* = 1.000, d = 0.001) and eccentric protocols (∆pre-post: *p* = 1.000, d = 0.128, and ∆pre-24 h-post: *p* = 1.000, d = 0.152). The CK increased slightly but statistically significantly after concentric cycling (∆pre-post: +3.05 ± 4.75%, *p* = 0.024, d = 0.024), while the increase nearly reached statistical significance after eccentric cycling (∆pre-post: +2.67 ± 4.92%, *p* = 0.055, d = 0.020). However, changes in CK levels 24-h after the protocols were no longer statistically significant in both the concentric (∆pre-24 h-post: 2.67 ± 16.40%, *p* = 0.720, d = 0.037), and the eccentric protocols (∆pre-24 h-post: 15.28 ± 31.84%, *p* = 0.341, d = 0.095).

**Figure 4:**
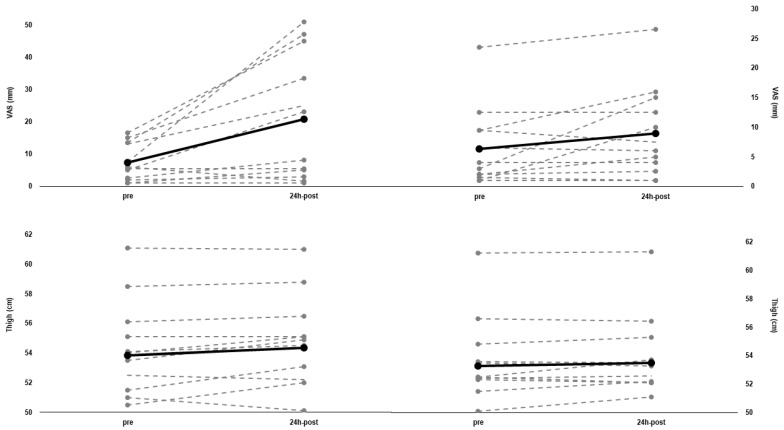
Individual values (*n* = 12) and mean values (solid line) for visual analogue scale (VAS) and thigh circumference from baseline (pre), to 24 h after (24 h-post) eccentric cycling (left panel) and concentric cycling (right panel).

VAS statistically increased 24-h after eccentric cycling (from: 7.39 ± 5.44 to 20.71 ± 18.48, *p* = 0.016, d = 2.969), while it nearly reached statistical significance 24-h after concentric cycling (from: 6.33 ± 6.37 to 8.92 ± 7.23, *p* = 0.065, d = 0.517). The thigh circumference remained unchanged 24 h after concentric cycling (∆pre-24 h-post: +0.44 ± 0.95%, *p* = 0.159, d = 0.073), but increased 24 h after eccentric cycling (∆pre-24 h-post: +0.97 ± 1.45%, *p* = 0.045, d = 0.152). Both VAS and thigh circumference changes are shown in Figure 4 on an individual basis.

### 3.3. Relationships between Parameters

An inverse statistical relationship between VO_2_peak and peak blood lactate concentrations were observed after the concentric protocol (r = −0.590, *p* = 0.043). In addition, a statistical association was observed between %ΔPP and %ΔKvert during the CMJ immediately (r = 0.879, *p* = 0.000), and 24 h after (r = 0.786, *p* = 0.002) the eccentric but not the concentric protocol. Some statistically significant associations were observed only in the eccentric condition for muscle damage markers: pre- to post- changes of BAG3 and MMP-13 (r = 0.74, *p* = 0.006), changes from pre- to 24 h-post-loading of BAG3 and MMP13 (r = 0.68, *p* = 0.015), and changes from pre to 24 h-post-loading of BAG3 and CK (r = 0.61, *p* = 0.036). No statistically significant associations were observed between ∆CK and ∆MMP13 at any time point. No other statistically significant associations were identified among the selected outcomes.

## 4. Discussion

This study aimed to describe and compare, for the first time, the internal and external loads of time-matched eccentric and concentric cycling protocols with 5 s “all out” efforts, and their effects on functional performance and selected markers of muscle damage, soreness, and swelling. As expected, the eccentric protocol exhibited lower cardiometabolic responses than the concentric one. However, both mean and peak power were statistically lower in the eccentric protocol, despite the sprint bouts being performed “all out” in both conditions. In addition, and contrary to our expectations, the eccentric protocol did not result in any relevant disturbances in functional performances. However, subjects experienced some lower-limb soreness accompanied by a slight muscle swelling 24-h following the eccentric protocol only. These findings are important, as they may demonstrate the suitability of similar eccentric protocols for future use with different populations. Similarly, although the cardiometabolic responses were greater during the concentric protocol, the values recorded during this protocol were also lower when compared to previous concentric HIT protocols [28], thus suggesting that both protocols are appealing candidates for HIT prescription in different populations.

The lower cardiometabolic responses during the eccentric protocol are in line with the findings of previous studies, where mean values for HR and blood lactate concentrations were even lower than that reported for concentric continuous low-intensity exercise [8,18]. Moreover, the present concentric protocol also exhibited very low HR and peak blood lactate values when compared to previous studies with concentric modified SIT protocols, probably because of the lower number of sprints (8 vs. 16 sprint bouts) and the longer recovery time (55 vs. 24 s) [28,36]. However, in the current study, subjects were not able to achieve greater peak power values in the eccentric than in the concentric sprint bouts. It is noteworthy that the values recorded in our study were also lower than those previously reported with peak values >1000 W [10]. However, factors related to cycling technique, ergometer characteristics, and the endurance training background of our subjects would explain discrepancies between studies. For instance, differences in sampling rate between ergometers, and in maximal cadences achieved during sprinting bouts in the concentric mode (>110 rpm), may limit comparisons [37,38]. Furthermore, the validity and reliability of peak power measures during short (<10 s) sprinting bouts may not be sufficient in some cases [39]. Despite these concerns, which are out of the scope of the present investigation, it is worth noting that the absolute values recorded during both the concentric and eccentric protocols were 2- and 3-fold higher than the PPO values recorded in the concentric cycling ramp test. While greater power outputs may be achieved with greater cadences in eccentric cycling (e.g., 100 rpm), this may imply, in turn, a more technical difficulty that may possibly be overtaken with even more familiarization sessions. It is of note that, a progressive increase in pedal cadences during eccentric cycling, as occurs in concentric cycling sprints, is technically impossible. Therefore, the appropriate cadences for eccentric sprinting for both healthy and clinical populations remain to be determined. Future studies should also consider the technical characteristics of ergometers (e.g., body position, sampling rate) for appropriate comparisons between contraction modes [26]. Meanwhile, the instruments and ergometers used in the current study fairly reproduce the possibilities of most training settings, therefore confirming the high ecological validity of our experiment.

In line with external load parameters, lower perceived exertion and more positive mood scores were also recorded during the eccentric sprint bouts, even though the difference between conditions was not statistically significant. To the best of our knowledge, the present study is the first to report RPE and FS scores for an eccentric HIT protocol. Previously, one study [11] reported similar RPE and enjoyment scores between different continuous and eccentric HIT protocols. Interestingly, the perceptual responses (i.e., RPE and FS) during the concentric protocol in the current study were better than that observed in previous studies [28]. The positive mood scores during the time-matched protocols, could be related to the lower neuromuscular activation and to the work-to-rest ratio [28]. Thus, both neuromuscular and glycolytic activation of the current protocols, may be the major factors for these specific responses. Further studies should manipulate these factors when looking for different perceptual responses, including the evaluation of other different psychological constructs (e.g., enjoyment). In addition, the low sRPE values for the current protocols reinforces the low loading experienced by subjects despite completing 8 “all out” efforts in both sessions, also allowing comparisons with other exercise modalities with a well-accepted method for training monitoring [40].

One surprising finding was the absence of functional performance impairments after both protocols, and more specifically after the eccentric protocol. While jumping height remained unaltered immediately after and 24 h post the eccentric protocol, an acute increase in jumping height was observed after the concentric one. Previous studies on concentric SIT protocols reported significant impairments in jump capacity associated with peripheral fatigue in long (i.e., 20 s) but not in very short sprints [28], while studies on submaximal eccentric cycling reported acute and delayed impairments in explosive force capacity associated with muscle damage [41]. However, this was not observed in the present study, despite the greater mechanical power values recorded. In addition, the jump potentiation [42] observed after the current concentric protocol is not surprising considering the reduced work-to-rest ratio when compared to a previous study with a modified SIT protocol utilizing more sprint bouts and shorter recovery intervals [28]. In this regard, it is interesting to note that the jumping height exhibited a tendency to increase immediately after the eccentric protocol. The non-significant reductions in Kvert that were correlated with the non-significant changes in PP in this condition may be reflecting some loss of eccentric force capacity that would be counteracted by an enhanced concentric phase of the impulse, as has been shown previously [43]. Meanwhile, the preservation of handgrip strength up to 24 h after the protocols would suggest the absence of important fatigue in the upper limbs associated to the cycling posture during “sprinting” bouts, which is also an important finding to be reported considering that most previous studies with eccentric cycling were performed in recumbent ergometers.

The limited changes of the previous loading parameters may be explained by a very low magnitude of blood derived EIMD indices. In fact, a statistical increase in CK concentrations was observed only immediately after the concentric protocol but the magnitude was very low (i.e., 232 U∙L^−1^) and it is likely that these changes are not of clinical significance. Similarly, the serum levels of BAG3 and MMP-13 remained statistically unaltered immediately after both protocols and at 24-h of recovery. The apparent absence of EIMD may well be related to the inclusion of two familiarization sessions with maximal and “all out” efforts before experimental sessions [44]. However, a low but statistical increase in muscle soreness and thigh circumference was observed 24-h following eccentric protocol. In fact, this is in line with previous reports utilizing different eccentric cycling bouts [12,18,45]. The absence of significant changes in BAG3 and MMP-13, in conjunction with the low muscle soreness and swelling only 24-h after the eccentric protocol may not be contradictory, as muscle damage and soreness are not necessarily related and may potentially be induced by different mechanisms [12,44].

When interpreting our findings, a limited validity of BAG3 and MMP-13 for measuring the acute muscle damage must be acknowledged. This is particularly attributed to a high biological variability, probably associated with their multiple functions for proteostasis during different phases of recovery from EIMD [24,46], but also because concentrations of these markers were not directly obtained from muscle biopsies as in previous studies [23,25]. However, our analysis indicated an acceptable reliability for basal values, indicating these markers to be relatively stable in serum. Moreover, the statistical associations between the changes in BAG3, MMP-13, and CK would reinforce the suitability of these markers and, at the same time, indicate these markers as potential targets for further studies evaluating EIMD after eccentric protocols. Therefore, future studies should explore the sensitivity of these specific markers to monitor different facets of muscle damage over time.

### Study Limitations

The time points selected for analyses were limited, therefore one could argue that more days may be required to better characterize the delayed responses of our protocols. Considering that we may have induced a RBE [44] by performing two familiarization sessions and likely, as a result, only minor EIMD symptoms were observed 24-h post-testing, we would not expect significant differences in most measures after 48–96 h. This assumption is further supported by the young age and high physical fitness of the subjects [47,48,49]. However, although some subjects verbally reported some low discomfort the days following familiarizations, we did not record any EIMD data in the laboratory, therefore future studies are needed to confirm this expected RBE. In addition, the use of thigh circumference for assessing muscle swelling is a possible limitation. Therefore, the significant change of this measure after the eccentric protocol should be interpreted with caution despite exhibiting the best reliability of all included parameters. Finally, it would be desirable to test the two protocols with athletes or patients who are in the target populations for this type of interventions. Thus, we encourage future studies to consider all these aspects with similar protocols, both in terms of acute responses but also chronic training adaptations.

## 5. Conclusions

To the authors’ knowledge, this is the first study comparing two time-matched cycling protocols of 5-s “all out” efforts in both concentric and eccentric modes. Lower cardiometabolic responses were observed in the eccentric protocol, however the concentric protocol also exhibited low cardiometabolic responses when compared to previous studies with concentric HIT. Both protocols were perceived similarly low. In addition, no negative effects on functional performance measures and markers of muscle damage were observed after both protocols, while minimal muscle soreness and swelling were observed only 24 h after the eccentric protocol. Taken together, these results suggest the appropriateness of these time-efficient and safe protocols to be used with different populations in future longitudinal interventions for both health and performance purposes. Thus, with very short (5 s) “all out” efforts during very short sessions (10 min), we have accumulated a very high amount of work (~60–80 kJ) but with much reduced perceptual loading and EIMD symptoms.

## Figures and Tables

**Table 1:** Physiological and mechanical responses of the cycling protocols.

Variable	Concentric Protocol (Mean ± SD)	Eccentric Protocol (Mean ± SD)	Between-Group (*p*-Value)	Cohen’s d
Mean HR (bpm)	129.7 ± 11.2	96.5 ± 12.2	<0.001	2.841
Mean HR (% HRmax)	68.8 ± 6.6	51.3 ± 5.7	<0.001	2.818
Peak HR (bpm)	154.3 ± 12.2	116.2 ± 14.1	<0.001	2.897
Peak HR (% HRmax)	81.8 ± 4.6	61.8 ± 6.6	<0.001	2.897
Mean Power (W)	137 ± 11	101 ± 15	<0.001	2.812
Mean Power (% PPO)	31.6 ± 2.8	23.3 ± 3.8	<0.001	2.493
Sprint Peak Power (W)	1112 ± 153	782 ± 112	<0.001	2.465
Sprint Peak Power (% PPO)	254.6 ± 22.9	179.9 ± 27.3	<0.001	2.966
Total Work (J)	82,824 ± 6350	60,602 ± 8904	<0.001	2.874
Peak Lactate (mmol·L^−1^)	4.9 ± 2.1	1.8 ± 0.6	<0.001	2.042

HR: heart rate, PPO: peak power output in the ramp test.

**Table 2:** Functional performance changes induced by the cycling protocols.

Variable	Concentric Protocol (Mean ± SD)			Eccentric Protocol (Mean ± SD)			Between-Groups (Cohen’s d)		
	Pre	Post	24-h	Pre	Post	24-h	Pre	Post	24-h
Jumping height (cm)	31.5 ± 5.0	33.3 ± 5.0 *	32.0 ± 4.7	30.8 ± 5.4	31.7 ± 5.1	31.5 ± 5.2	0.135	0.317	0.101
PP (W·kg^-1^)	26.7 ± 4.0	27.9 ± 3.8 *	26.8 ± 3.8	26.7 ± 4.6	27.0 ± 4.6	26.3 ± 4.0	0.000	0.213	0.128
Kvert (N·m^-1^·kg^-1^)	56.8 ± 7.1	57.7 ± 8.4	57.1 ± 8.6	59.5 ± 12.9	56.9 ± 10.6	55.2 ± 8.6	0.259	0.084	0.221
Handgrip strength (kgf)	53.8 ± 5.3	53.8 ± 3.7	53.6 ± 4.7	55.7 ± 6.3	55.5 ± 5.9	53.7 ± 5.0	0.326	0.345	0.021

PP: peak power; Kvert: normalized vertical stiffness. * statistical change to pre, *p* < 0.05.

**Table 3:** Baseline values of markers of muscle damage assessed prior to each loading.

Variable	Concentric Protocol (Mean ± SD)	Eccentric Protocol (Mean ± SD)	ICC	CV
BAG3 (ng∙mL^−1^)	1.9 ± 1.1	2.0 ± 1.3	0.952	8.3%
MMP-13 (pg∙mL^−1^)	1281 ± 2285	1087 ± 1922	0.980	19.4%
CK (U∙L^−1^)	228 ± 154	215 ± 133	0.942	8.5%
VAS (mm)	6.3 ± 6.4	7.4 ± 5.4	0.642	26.1%
Thigh circumference (cm)	53.3 ± 3.1	53.9 ± 3.4	0.977	0.8%

BAG3: BCL2-associated athanogene 3; MMP-13: collagenase matrix metalloproteinase 13; CK: creatine kinase; VAS: visual analogue scale.

## Data Availability

Not applicable.
